# Uptake and utilization of nitrogen, phosphorus and potassium as related to yield advantage in maize-soybean intercropping under different row configurations

**DOI:** 10.1038/s41598-020-66459-y

**Published:** 2020-06-11

**Authors:** Yuanfang Fan, Zhonglin Wang, Dunping Liao, Muhammad Ali Raza, Beibei Wang, Jiawei Zhang, Junxu Chen, Lingyang Feng, Xiaoling Wu, Chunyan Liu, Wenyu Yang, Feng Yang

**Affiliations:** 1 College of Agronomy, Sichuan Agricultural University, Chengdu, 611130 People’s Republic of China; 2 Sichuan Engineering Research Center for Crop Strip Intercropping System, Chengdu, 611130 People’s Republic of China; 3 Key Laboratory of Crop Ecophysiology and Farming System in Southwest, Ministry of Agriculture, Chengdu, 611130 People’s Republic of China

**Keywords:** Plant sciences, Plant ecology

## Abstract

Intercropping advantage occurs only when each species has adequate time and space to maximize cooperation and minimize competition between them. A field experiment was conducted for two consecutive years between 2013 and 2014 to investigate the effects of maize and soybean relay strip intercropping systems on the uptake and utilization of nitrogen, phosphorus, and potassium. The treatments included “40:160” (T1, maize narrow and wide row spacing of 40 and 160 cm, where two rows of soybean with a 40 cm row were planted in the wide rows. The area occupation ratio of maize and soybean both were 50% of the every experimental block), “80:120” (T2, maize narrow and wide row spacing of 80 and 120 cm, the soybean planting was the same as T1 treatment. The area occupation ratio of maize and soybean were 60% and 40% of the every experimental block), “100:100” (T3, one row of maize and one row of soybean with a 100-cm row. The area occupation ratio of maize and soybean was the same as T1 treatment), sole cropping of maize (CK1, The area occupation ratio of maize was 100% of the every experimental block), and sole cropping of soybean (CK2, The area occupation ratio of soybean was 100% of the every experimental block). The results show that, compared with the sole cropping system (sole maize), the economic yields in T1, T2, and T3 treatments increased by 761, 536, and 458 kg·ha^−1^, respectively, and the biological yields increased by 2410, 2127, and 1588 kg·ha^−1^. The uptake and utilization of nitrogen, phosphorus, and potassium in T1, T2, and T3 treatments were significantly higher than those in sole crops, and the nutrient advantage is mainly due to nutrient uptake rather than nutrient use efficiency. The land equivalent ratio values in T1, T2, and T3 treatments were 1.43, 1.32, and 1.20, respectively. In particular, the economic and biological yield in T1 treatment exhibited potential as an intercropping pattern.

## Introduction

The world’s agriculture is currently facing a new challenge and the global grain security problem persists^1^. Such problems include ever-growing population, cultivated land conversion into urban and industrial construction, and climate change^2^. A major challenge is to enable biodiversity conservation while addressing the issue of food security^3^. Intercropping can improve the multiple cropping index and land utilization rate and ensure the high and stable yields of crops to achieve sustainable agricultural development^4^.

Intercropping involves two or more crop species growing together and coexisting for a specific time. Recent research indicated that cassava grown in America and Africa is typically intercropped^5^. Legume–cereal intercropping, especially maize–bean intercropping, is conventional throughout East and Southern Africa^6^. Intercropping is also practised in temperate zones. In northwestern China, the different kinds of intercropping are wheat–maize^7^, maize–soybean^8^, sunflower–soybean^9^, and maize–pea^10^.

One of the benefits of intercropping is efficient resource use through niche differentiation and complementarity^11^. Intercropping also leads to higher crop yield and productivity than planting single crop^12^. Intercropping between grasses and legumes not only results in high yield but also promotes the uptake of nitrogen by crops^3,4,13^. For example, in intercropping maize and legumes, the former uses nitrogen from the soil for growth and the latter relies on atmospheric N_2_ fixation for growth^14^. Previous studies reported that the transfer of fixed nitrogen from Vicia faba to wheat is due to the nitrogen fixation of legumes and the large nitrogen requirement of grass in the case of nitrogen deficiency^15^. Intercropping is not only beneficial to increase the nitrogen uptake of crops but also promotes the uptake of phosphorus. The interspecific promotion of phosphorus is prominent when grasses are intercropped with legumes, especially, faba bean^16^, white lupins^17^, and chickpeas^18^. Potassium is mainly present in the form of ions in soil and is transferred by diffusion. The uptake and utilization of potassium by crops is related to fertilizer types and their genetic characteristics. Some intercropping systems exert a promoting effect on potassium^19,20^.

Intercropping effective utilization of resources by crop intensification both in space dimensions^21^. For instance, improved and efficient use of the light environment is possible with two or more species in the same land. Due to the roots being at different depths and rooting patterns create the space dimension of intercropping. The crop nutrient requirement in varies among different crops due to their leaf morphology, photosynthesis and growing conditions differences^22^. Intercropping advantage occurs in only when each species has adequate time and space to maximize cooperation and minimize competition between them.^23^ Therefore, Changing the hierarchies and spatial patterns in plant populations may influence the productivity of the intercropping system.

The present study aimed to: (1) analyze the yield advantage of maize–soybean intercropping system and comprehensively evaluate its nutrient absorption and utilization efficiency; (2) determine the quantitative relationship of the land equivalent ratio (LER) to nutrient absorption and utilization efficiency and analyze the contribution of nutrient absorption and utilization efficiency to the yield advantage of maize and soybean intercropping; and (3) through different row configurations, the optimal configuration of system yield and benefits can be obtained in maize–soybean relay strip intercropping system.

## Results

### Crop yield and composition

The economic and biological yields of the maize-soybean intercropping system are larger than those of the sloe cropping system (Fig. 3). The results show that, compared with the sole maize, the economic yields (the sum of maize and soybeans) in T1, T2, and T3 treatments increased by 761, 536, and 458 kg·ha^−1^, respectively (Fig. 3a,b), and the biological yields (Fig. 3c,d) increased by 2 410, 2127, and 1588 kg·ha^−1^ (average of years 2013 and 2014). Two years of data showed no significant differences in all treatments on the effective panicle, thousand kernel weight, kernel number, and yield (Table 1). The yield of maize under T1, T2, and T3 treatments was lower than that of CK1. The yield of maize under the T1, T2, and T3 treatments was reduced by 10.9%, 5.9%, and 0.4% compared with CK1, respectively (average of years 2013 and 2014). Soybean yield was significantly affected by the narrow row spacing of maize in the maize–soybean relay intercropping system (Table 1). The yield of soybean in T1, T2, and T3 treatments was significantly decreased by 12.1%, 44.9%, and 72.7% compared with CK2_,_ respectively (average of years 2013 and 2014). The narrow row planting pattern did not significantly affect the 100-grain weight and the number of seeds in soybeans. The reduction in the yield of soybean was mainly caused by the decrease in the number of effective plants and the number of pods per plant.

**Figure 1:**
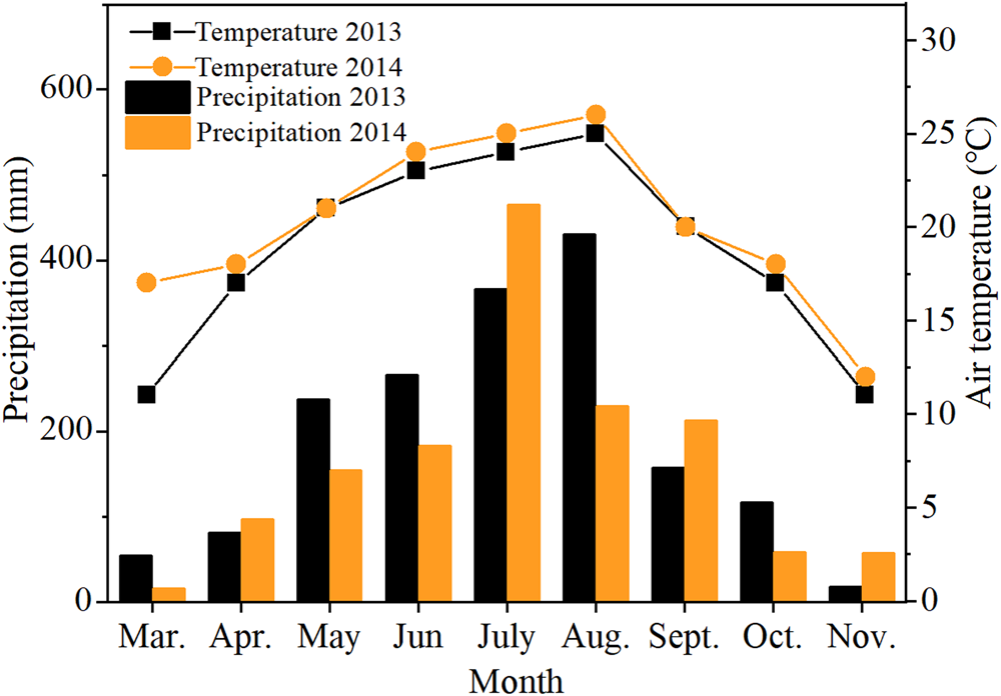
Monthly mean precipitation and temperature during the growth stage of the intercrops in 2013 and 2014.

**Figure 2:**
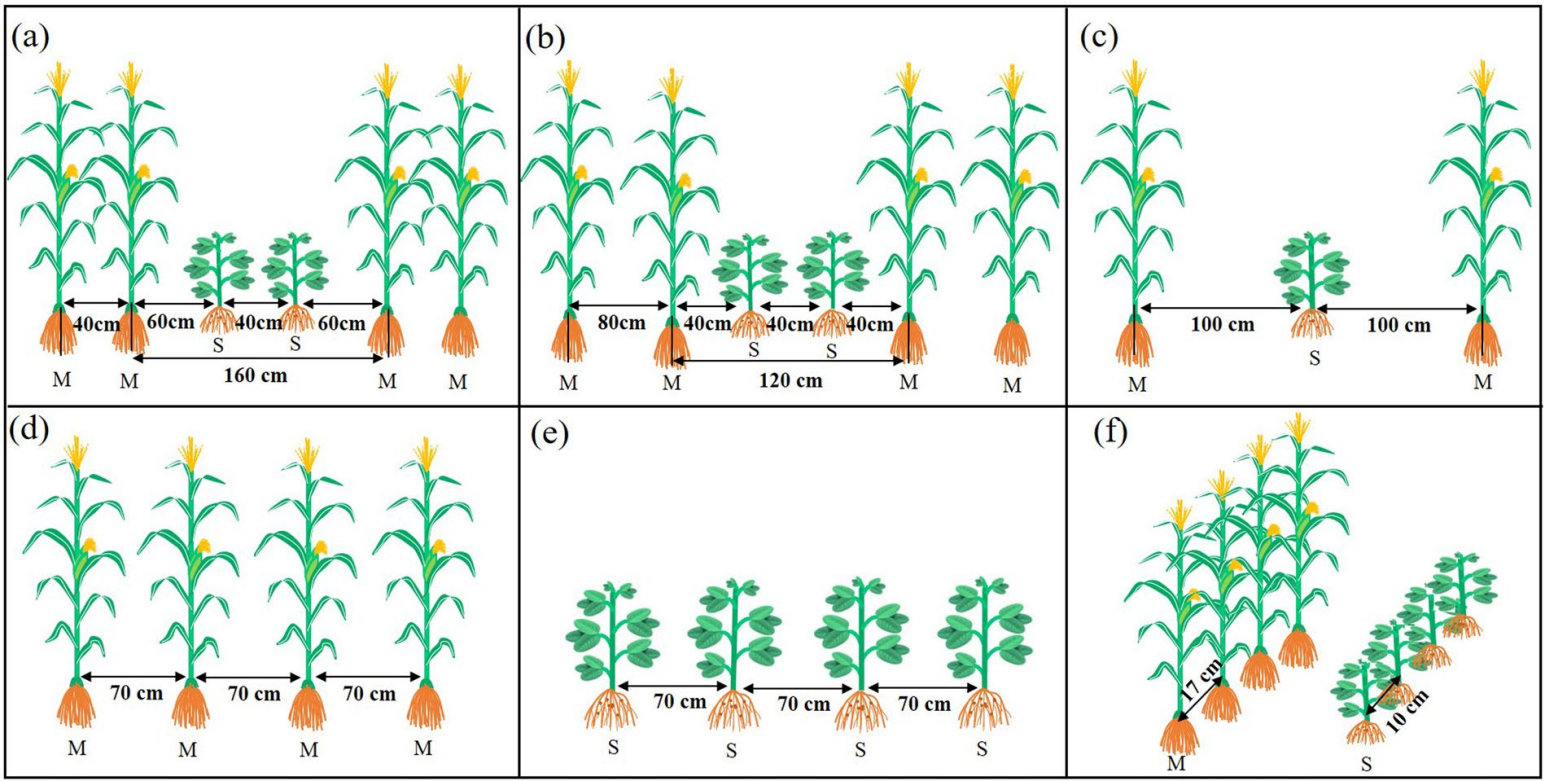
The layout of different narrow row configuration between maize (M) and soybean (S). The bandwidth of intercropping was always 200 cm, and soybean was planted in a wide row of 40 cm. The narrow row spacing of maize was 40 cm (**a**, T1) and 80 cm (**b**, T2); at the same time, the distances between maize row and soybean row were 60 and 40 cm, respectively. 100 cm (**c**, T3) spacing configuration and sole maize (**d**, CK1), soybean (**e**, CK2) as a control. Sole crops have the same row spacing of 70 cm. The plant spacings of maize and soybean were 17 and 10 cm (**f**), respectively.

**Figure 3:**
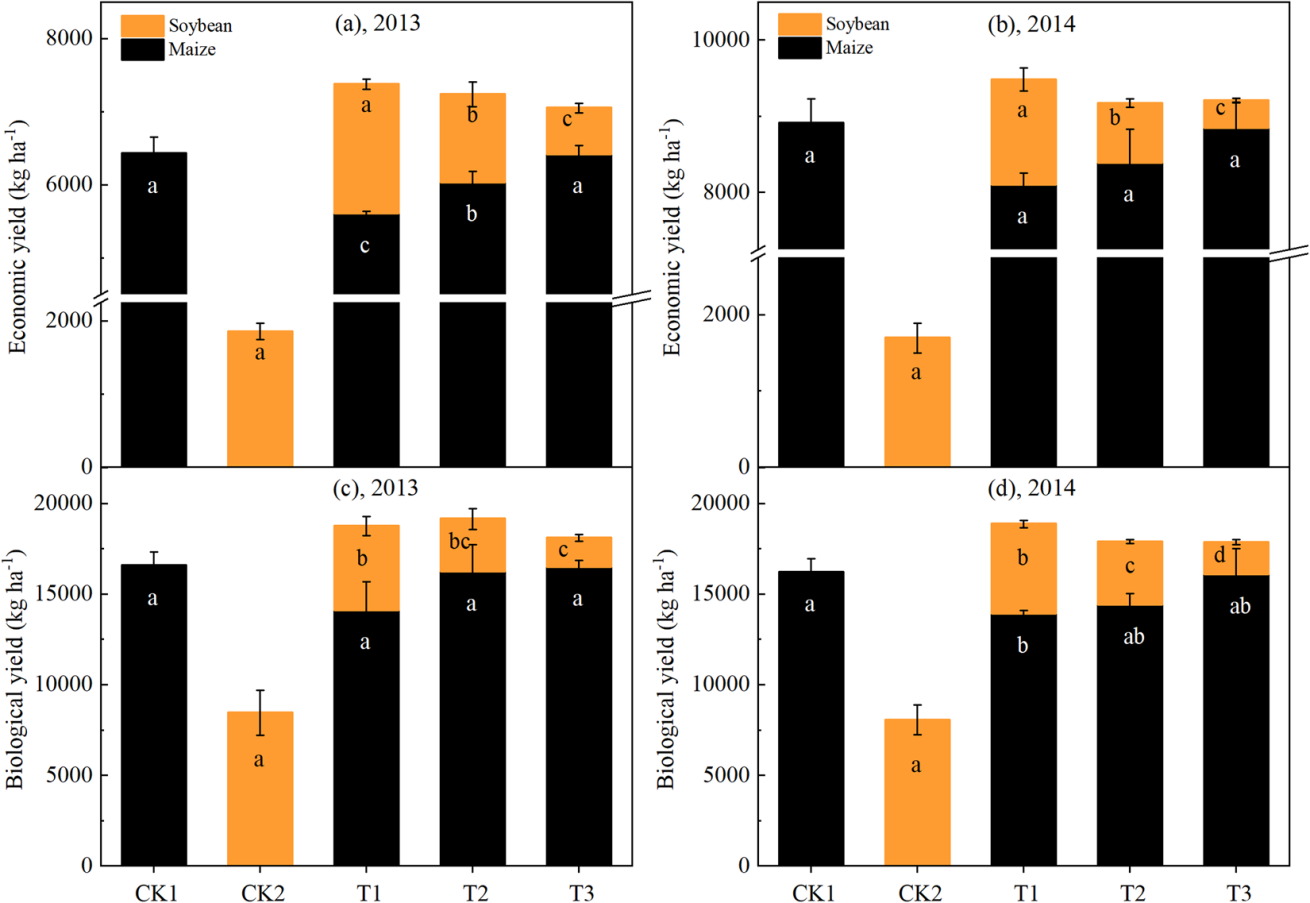
The economic yield and biological yield of maize and soybean are grown as sole cropping and relay intercropping system. The CK1 and CK2 represent sole cropping system of maize and soybean, respectively. The T1 (40 cm: 160 cm), T2 (80 cm: 120 cm), and T3 (100 cm: 100 cm) represent the different narrow row configuration under relay-intercropping system. Means are averaged over three replicates. Bars show ± standard errors, (n = 3). Within a bar, different lowercase and same letters (white letters: maize; black letters: soybean) show a significant and non-significant difference (p ≤ 0.05) between treatments.

**Table 1:** Effect of row configurations on the yield and yield components of maize and soybean under sole soybean cropping system and relay intercropping system in 2013 and 2014.

	Year	2013				2014			
	Treatment	CK	T1	T2	T3	CK	T1	T2	T3
Maize	Effective ear (×10^3^ Plant ha^−1^)	50.19a	51.11a	50.67a	53.34a	46.27a	45.56a	48.69a	47.78a
	Thousand kernel Weight (g)	249.1a	229.6a	225.6a	225.1a	250.3a	266.2a	267.8a	265.9a
	kernel number	505.0a	479.3a	511.1a	537.3a	689.9a	675.2a	692.1a	701.3a
	Yield (kg ha^−1^)	6429a	5612c	6040b	6422a	8908a	8109a	8397a	8850a
Soybean	Effective number (10^3^ plant ha^−1^)	74.17ab	79.49a	85.04a	61.70b	73.00a	78.93a	67.81a	55.58b
	Number of pods (plant^−1^)	87.94a	73.03a	45.81b	34.68b	68.00a	66.98a	45.61b	24.98c
	Number of seed (pod^−1^)	1.61a	1.69a	1.71a	1.63a	1.72a	1.53a	1.51a	1.57a
	100-seed mass (g)	17.74a	18.00a	17.90a	18.10a	19.38a	17.10a	16.50a	16.20a
	Yield (kg ha^−1^)	1856a	1764a	1197b	628c	1693a	1373a	775b	353c

The T1, T2, and T3 represent “40:160” (maize narrow and wide row spacing of 40 and 160 cm, where two rows of soybean with a 40-cm row were planted in the wide rows), “80:120” (maize narrow and wide row spacing were 80 and 120 cm, the soybean planting was the same as T1 treatment), “100:100” (one row of maize and one row of soybean with a 100-cm row) respectively. Values followed by different letters within the same line show a significant difference between different treatments (P < 0.05).

### Nutrient uptake

As shown in Table 2, the nitrogen uptakes under T1, T2, and T3 treatments were 226.14, 211.42, and 189.87 kg ha^−1^, respectively, and those of weighted mean for sole crops were 149.59, 153.04, and 149.59 kg ha^−1^ (average of the year 2013 and 2014). The nitrogen uptake under the T1, T2, and T3 treatments increased by 51.4%, 37.7%, and 26.4%, respectively, compared with the sole crops. Similarly, the phosphorus uptake under T1, T2, and T3 treatments increased by 57.0%, 37.0%, and 39.6%, respectively, compared with the sole crops. The potassium uptake in T1, T2, and T3 treatments increased by 40.9%, 31.0%, and 16.9%, respectively, compared with the sole crops. The uptake of nitrogen, phosphorus, and potassium in T1 treatment was significantly better than that in T2 and T3 treatments. We found that the concentrations of nitrogen, phosphorus and potassium in various organs of soybean were reduced considerably under the intercropping system, compared to the sole cropping system, but had almost no difference on maize (Fig. S1).

**Table 2:** Nutrient uptake between maize–soybean relay intercropping system and sole cropping systems at maturity in 2013 and 2014.

Year	Nutrient	Treatment	Intercrops (kg ha^−1^)	Weight means for sole crops (kg ha^−1^)	Changes in nutrient uptakes by intercrops relative to sole crops (△NU, △PU, △KU)%
2013	Nitrogen	T1	234.61a	158.44a	48.07
		T2	235.91a	163.06a	44.68
		T3	214.37b	158.44a	35.30
	Phosphorus	T1	48.10a	33.70a	42.73
		T2	48.74a	34.01a	43.31
		T3	43.43b	33.70a	28.87
	Potassium	T1	194.84a	134.91a	44.42
		T2	186.45b	134.82a	38.30
		T3	163.00c	134.91a	20.82
2014	Nitrogen	T1	217.67a	140.74a	54.66
		T2	186.92b	143.02a	30.70
		T3	165.37c	140.74a	17.50
	Phosphorus	T1	47.33a	27.64b	71.24
		T2	39.72c	30.40a	30.66
		T3	41.56b	27.64b	50.36
	Potassium	T1	208.29a	151.67b	37.33
		T2	194.90b	157.67a	23.61
		T3	171.42c	151.67b	13.02

The T1, T2, and T3 represent “40:160” (maize narrow and wide row spacing of 40 and 160 cm, where two rows of soybean with a 40- cm row were planted in the wide rows), “80:120” (maize narrow and wide row spacing were 80 and 120 cm, the soybean planting was the same as T1 treatment), “100:100” (one row of maize and one row of soybean with a 100-cm row) respectively. Values followed by different letters within the same column show a significant difference between different treatments (P < 0.05).

### Nutrient use efficiency

The study indicated that high utilization efficiency of nitrogen, phosphorus, and potassium in the intercropping system. The nitrogen, phosphorus, and potassium utilization efficiencies in T1, T2, and T3 treatments were slightly higher than those of sole crops (Table 3). The utilization efficiency of nitrogen, phosphorus, and potassium by the intercropping systems increased with increasing narrow row spacing for maize. The nitrogen, phosphorus, and potassium nutrient utilization efficiency in T3 treatment increased by 9.9%, 13.7%, and 19.6%, respectively, in 2013. The nitrogen and potassium utilization of T3 treatment increased by 27.9%, 13.3%, and 34.1%, compared with the sole crops. The nutrient use efficiency of nitrogen, phosphorus and potassium in various organs of maize and soybean were almost no difference under the intercropping system, compared to the sole cropping system (Fig. S2).

**Table 3:** Nutrient use efficiency between maize–soybean relay intercropping system and sole cropping systems at maturity in 2013 and 2014.

Year	Nutrient	Treatment	Intercrops (kg kg^−1^)	Weight means for sole crops (kg kg^−1^)	Changes in nutrients efficiency by intercrops relative to sole crops (△NU,△PU,△KU)%
2013	Nitrogen	T1	79.87b	76.84b	3.94
		T2	81.18ab	79.73a	1.82
		T3	84.42a	76.84b	9.86
	Phosphorus	T1	389.55b	366.36b	6.33
		T2	392.89b	387.09a	1.50
		T3	416.69a	366.36b	13.74
	Potassium	T1	96.18 c	92.82b	3.62
		T2	102.71b	98.90a	3.85
		T3	111.03a	92.82b	19.62
2014	Nitrogen	T1	86.68 c	84.44a	2.65
		T2	95.71b	88.87a	7.70
		T3	108.02a	84.44a	27.93
	Phosphorus	T1	467.95b	398.57 c	17.41
		T2	478.41b	450.38a	6.22
		T3	487.14a	429.81b	13.34
	Potassium	T1	90.58b	77.74a	16.52
		T2	91.79b	80.04a	14.68
		T3	104.21a	77.74a	34.05

T1, T2, and T3 represent “40:160” (maize narrow and wide row spacing of 40 and 160 cm, where two rows of soybean with a 40- cm row were planted in the wide rows), “80:120” (maize narrow and wide row spacing were 80 and 120 cm, the soybean planting was the same as T1 treatment), “100:100” (one row of maize and one row of soybean with a 100-cm row) respectively. Values followed by different letters within the same column show a significant difference between different treatments (P < 0.05).

### Contribution of nutrient uptake and utilization efficiency to the advantages of the system

The LER of the maize–soybean relay strip intercropping system decreased with increasing narrow row distance of maize (Table 4). The LER values in T1 (1.43), T2 (1.32), and T3 (1.20) treatments were greater than 1, showing the advantages in the intercropping system. The advantage of intercropping was mainly due to the increase in nutrient uptake rather than the improvement of nutrient use efficiency (Table 4). The contribution of nitrogen, phosphorus, and potassium uptake factors in T1, T2, and T3 treatments was positive (nitrogen uptake factor was 0.48–0.18, phosphorus uptake factor was 0.46–0.20, and potassium uptake factor was 0.41–0.11; an average of the year 2013 and 2014). However, the utilization and interaction factors slightly differed between 2013 and 2014. The nutrient utilization factors under the T1, T2, and T3 treatments were negative in 2013. The nitrogen, phosphorus, and potassium nutrient utilization factors of the T1, T2, and T3 treatments in 2014 increased with increasing narrow row spacing, and the nutrient utilization factors of all treatments were all positive, except for the nitrogen utilization factor in T1 treatment.

**Table 4:** Contribution of uptake, utilization, and interaction factors to the intercropping advantage.

Year	Nutrient	Treatment	LER	$(1+{a}_{m}+{a}_{s})$	$({e}_{m}+{e}_{s})$	$({a}_{m}{e}_{m+}{a}_{s}{e}_{s})$
2013	Nitrogen	T1	1.40	0.43	−0.03	0.00
		T2	1.32	0.39	−0.10	0.03
		T3	1.18	0.23	−0.03	−0.02
	Phosphorus	T1	1.34	0.41	−0.03	−0.04
		T2	1.32	0.41	−0.07	−0.02
		T3	1.19	0.25	−0.05	−0.01
	Potassium	T1	1.40	0.45	−0.05	0.00
		T2	1.32	0.38	−0.09	0.03
		T3	1.18	0.21	−0.01	−0.02
2014	Nitrogen	T1	1.47	0.53	−0.09	0.03
		T2	1.32	0.30	0.00	0.02
		T3	1.22	0.12	0.10	0.00
	Phosphorus	T1	1.48	0.51	0.02	−0.05
		T2	1.32	0.21	0.19	−0.08
		T3	1.21	0.14	0.13	−0.06
	Potassium	T1	1.47	0.36	0.15	−0.04
		T2	1.32	0.21	0.14	−0.03
		T3	1.21	0.01	0.28	−0.08

T1, T2, and T3 represent “40:160” (maize narrow and wide row spacing of 40 and 160 cm, where two rows of soybean with a 40- cm row were planted in the wide rows), “80:120” (maize narrow and wide row spacing were 80 and 120 cm, the soybean planting was the same as T1 treatment), “100:100” (one row of maize and one row of soybean with a 100-cm row) respectively. LER: the land equivalent ratio; (1 + a_m_ + a_s_): uptake factor; (e_m_ + e_s_): utilization factor; (a_m_e_m_ + a_s_e_s_): interaction factor.

## Discussion

### Effect of narrow row configuration on crop yield and composition

Intercropping is a commonly used agronomic practice in many countries because it can use nutrient, light and water resources efficiently and enhance crop yield^24^. The total area planted with maize–soybean intercropping exceeded 330,000 ha in the southwest of China^25,26^. Previous studies reported that reasonable group structure could reduce individual competition and reduce illumination loss, producing a conducive environment for the growth and development of individual crop and the full utilization of resources and improving the crop productivity^27^. In this study, the total economic and the total biological yield in T1, T2, T3 treatments under maize-soybean relay intercropping system increased compared to sole cropping system (Fig. 3). The growth and yield formation of maize were slightly affected by the crop group structure. The narrow row configuration primarily affected the maize yield through the changes in effective ear and kernel number, consistent with the findings of Liu *et al*. (2018). Soybean yield was mainly affected by the variations in maize and soybean spacing when the distance between them was reduced from T1 treatment to T3 treatment; the soybean yield decreased compared with that in CK2 (Table 1, Fig. 3), causing severe production cuts. Among the soybean yield components, the number of pods per plant and the number of effective plants were significantly affected by the narrow row configuration (Table 1). The spacing between the maize and soybean was reduced, thus decreasing the effective photosynthetic radiation (PAR) of soybean plant canopy^28^. The PAR at the top of soybean canopy positively affected the *P*_*n*_ and yield of intercropped soybean^8^. Therefore, when the distance between the maize and soybean was reduced from T1 treatment to T3 treatment, to the PAR, the *P*_*n*_ of intercropped soybean and the yield of soybean decreased.

### Effect of narrow row configuration on the nutrient uptake and utilization of the system

The biological basis of crop yield advantage in crop nutrition is mainly the increase of nutrient uptake and use efficiency^19^. The system nutrient advantage not only benefits from the increase in nutrient uptake but also nutrient transfer and utilization efficiency^29^. The high nutrient uptake under the intercropping system was superior to that under the sole cropping system, resulting in increased dry matter accumulation and yield. In our experiment, the nitrogen, phosphorus, and potassium uptakes of the intercropping system were higher than those of sole crops. The uptake under T1 treatment was the highest, which decreased with an increasing narrow spacing of maize (Table 2). Plants that compete for soil and fertilizer nitrogen in intercropping can enhance the ability of weakly competitive legumes to fix atmospheric nitrogen. The distance between maize and soybean decreased with increasing narrow distance of maize, thereby increasing the interspecific competition between maize and soybean. The ability of maize to compete for nitrogen in maize–soybean intercropping is stronger than that of soybeans^30^. The intercropped maize can absorb more nitrogen, whereas the soil nitrogen level in the soybean root zone is reduced or experiences nitrogen deficiency. The concentrations of nitrogen, phosphorus and potassium in various organs of soybean in T1, T2, and T3 treatments were significantly reduced under the intercropping system, compared to sole soybean, but had almost no difference on maize (Fig. S1). Nitrogen deficiency contributes to the improvement of nitrogen fixation capacity of legume crops, resulting in a significant increase in nitrogen uptake throughout the system. Besides, nitrogen fixed in legumes in legume/non-legume intercropping systems can be transferred to non-legumes and used by non-legume crops^31^. This phenomenon may also be one of the mechanisms of increased nitrogen uptake in intercropping. These results were obtained in the study of rye–pea^32^ and maize–peanut intercropping systems^10^.

The phosphorus, potassium uptake, and utilization efficiencies in T1, T2, and T3 treatments were higher than those of sole crops (Tables 2 and 3). The phosphorus and potassium uptake in T1 treatment was superior to those under T2 and T3 treatment. The phosphorus and potassium utilization efficiency of T3 treatment was the highest (average of the year 2013 and 2014). Although intercropping increased the amount of phosphorus and potassium absorbed, the uptake of nutrients in the system was affected by the row spacing configuration. Zhang (2016) pointed out that the roots are pivotal factors that control phosphorus uptake and improve the productivity of the faba–maize cropping systems (Zhang *et al*., 2016). Canopy cover also plays a key role in controlling the nutrient uptake for crops and boosting the productivity of intercropping systems^33,34^. In this study, when the narrow row spacing was increased, the spacing of the maize and soybean became small, which increased the canopy cover in soybean canopy. The increased canopy cover reduced the solar radiation reaching the soil surface and the temperature^35^. Increased canopy cover could increase N and P solubilization and reduce their loss^34^. This condition may increase the nutrient uptake in the maize and soybean intercropping system, thereby increasing the yield of this system. By contrast, intercropping inhibits the uptake of nitrogen, phosphorus, and potassium by plants, thereby inhibiting their growth^36^. These differences may be due to planting different intercropping crop differences. In conclusion, the maize and soybean relay strip intercropping system has a good promoting effect on nutrient uptake, which is a planting mode that is worthy of popularization and application in high-yield and high-efficiency modern agriculture.

### Contribution of uptake and utilization efficiency to intercropping advantage

An advantage in the intercropping system mainly depends on the contribution of nutrient uptake, utilization, and interaction factors based on crop nutrition. Two interesting features of nutrient utilization by crops in intercropping were noted from this study. First, the uptake factors of nitrogen, phosphorus, and potassium nutrients in the intercropping of maize and soybean had a positive contribution to the intercropping advantage (0.11–0.48), some of the nutrient utilization factor and interaction factors in this intercropping system were negative (Table 4). The contribution of the utilization factor to the yield advantage is usually much smaller than that of the uptake factor^19^. Hence, nitrogen, phosphorus, and potassium utilization efficiency show that in each treatment, the uptake factor mainly contributed towards the increase in LER for this intercropping system. The nitrogen, phosphorus, and potassium utilization factors were negative (2013), but the two-year average values of nitrogen, phosphorus, and potassium factors were mostly positive. Therefore, the yield advantage was primarily due to the higher total uptake of nutrients by the intercrops compared when they were grown in sole crops. The nitrogen, phosphorus and potassium nutrient dominance of the system decreased with increasing narrow row spacing. These results indicate that the uptake of T1 treatment was superior to that of the two other treatments. Second, the LER under T1, T2, and T3 treatments were greater than 1, showing the advantages of the maize–soybean relay strip intercropping system. The increased LER in relay strip intercropping is mainly due to the density-adding and configuration advantages^37^. The configuration advantage includes border row and complementary spatial advantages^8^. In this study, the LER of the maize–soybean relay strip intercropping system decreased with increasing narrow row distance of maize (Table 4). The LER of T1 treatment was more than that of T2 and T3 treatments, similar to previous literature reports^37^.

## Conclusion

Narrow row configuration affected the yield and yield component of maize and soybean in intercropping systems. Soybean yield was significantly decreased by the narrow row spacing of maize in the maize-soybean relay intercropping system, but maize yield was less affected. The total economic and the total biological yield in T1, T2, T3 treatments under maize-soybean relay intercropping system increased compared to the sole cropping system. The LER of all narrow row configurations were over 1.0, showing the high yield of the three-row configurations. The LER of the maize–soybean relay strip intercropping system decreased with an increasing row spacing of maize narrow rows, and the LER value in T1 treatment was the highest (1.43) among all treatments. Hence, T1 treatment had the optimal row spacing configuration. The maize–soybean relay strip planting system significantly increased the nutrient uptake of nitrogen, phosphorus, and potassium, but the effect on nutrient utilization efficiency was not noticeable. Therefore, the nutrient advantage was mainly due to the increased nutrient uptake rather than the nutrient use efficiency. Increasing the nutrient use efficiency is necessary for improving the yield advantage from nutrient uptake in intercropping.

## Materials and methods

### Experimental site

Two field experiments were conducted in 2013–2014 at the University Farm of Sichuan Agricultural in southwest China (29°54′N, 102°51′E) by using loam soil (typic purple soil) with the following properties: pH of 7.2, organic matter content of 24.3 g kg^−1^, total N of 1.3 g kg^−1^, total P of 1.1 g kg^−1^, total K of 15.4 g kg^−1^, available N of 298 mg kg^−1^, available P of 34.1 mg kg^−1^, and available K of 165.2 mg kg^−1^. The weather conditions (air temperature and monthly rainfall) during the growth stage of the intercrops in 2013 and 2014 are shown in Fig. 1.

### Experimental design

A relay intercropping system of maize and soybean was used in the field experiments. Two rows of maize were alternated with two rows of soybean. Soybean was grown in the wide rows of maize. Different row configurations were adopted to analyze the nutrient utilization and yield of maize and soybean intercropping. The five different row configurations treatments are shown in Fig. 2 and include the following: “40:160” (T1, maize narrow and wide row spacing of 40 and 160 cm, where two rows of soybeans with a 40 cm row were planted in the wide rows. The area occupation ratio of maize and soybean both were 50% of the every experimental block), “80:120” (T2, maize narrow and wide row spacing of 80 and 120 cm, the soybean planting same as T1 treatment. The area occupation ratio of maize and soybean were 60% and 40% of the every experimental block), “100:100” (T3, 100 cm row of maize and one row of soybean. The area occupation ratio of maize and soybean both were 50% of the every experimental block), sole cropping of maize (CK1, The area occupation ratio of maize was 100% of the every experimental block), and sole cropping of soybean (CK2, The area occupation ratio of soybean was 100% of the every experimental block).

In 2013, maize was sown on 28 March and harvested on 22 August, whereas soybean was planted on 17 June and harvested on 28 October. In 2014, maize was sown on 26 March and harvested on 20 August, whereas soybean was sown on 15 June and harvested on 26 October. We used the maize variety “Chuandan418” and the soybean variety “Nandou12” in both years. The plant densities of maize and soybean for the sole crops and intercrops were 60,000 and 100,000 plants ha^−1^, respectively. A randomized complete block design was used with three replicates.

All plots were treated with a basal fertilizer application. Total fertilizer is applied throughout the growing period of maize, the treatments comprised basal nitrogen at 255 kg ha^−1^ as urea, phosphorus at 600 kg ha^−1^ as calcium superphosphate, and potassium at 150 kg ha^−1^ as potassium chloride. At the seedling, jointing, and bell-mouthed stages of maize, the second dose of nitrogen was applied as fertilizer at 75 kg ha^−1^, with 150 kg ha^−1^ urea and 750 kg ha^−1^ ammonium bicarbonate^38^. At the sowing, seedling and flowering stages of soybean, nitrogen at 75 kg ha^−1^,135 kg ha^−1^ and 75 kg ha^−1^ as urea, phosphorus at 450 kg ha^−1^ as calcium superphosphate, and potassium at 60 kg ha^−1^ as potassium sulfate were applied^39^.

### Grain yields of maize and soybean

Actual and theoretical yields were determined in the R6 stage (maturity) of maize. The number of effective plants, 1,000-grain weight, and grain number per ear was investigated, and the grain was dried to a moisture content of approximately 14%. The yield was determined in the R8 stage (full maturity) of soybean. The number of effective plants, pods per plant, and pods per pod and 100-grain weight was determined, and grain was dried to a moisture content of approximately 12%.

### Nutrient content

The dried samples of the plants at maturity were separated into roots, stems, and leaves and powdered using the FW100 powder machine by passing through a 60-mesh sieve. Samples were weighing 0.15–0.2 g and boil with H_2_O_2_–H_2_SO_4_ combined by the boiled method. The cooked sample was diluted with water to 100 ml and mixed. After filtering the sample solution, the contents of nitrogen and phosphorus were measured by a flow analyzer (CAF, AA3, Germany) and the content of phosphorus was determined by PerkinElmer-AA400 flame atomic uptake spectrometry.

### Nutrient uptake

The nutrient uptake of intercropping was determined relative to the sole system. For example, nitrogen is calculated as follows:

1 $$\Delta NU \% =\{[N{U}_{ic}/({F}_{m}\times N{U}_{sm}+{F}_{s}\times N{U}_{ss})]-1\}\times 100 \% $$

*NU*_*ic*_ is the total nitrogen uptake of maize and soybean in intercropping. *NU*_*sm*_ and *NU*_*ss*_ are the nitrogen uptake of sole maize and sole soybean, respectively. *F*_*m*_ and *F*_*s*_ are the ratios of maize and soybean in intercropping, respectively. The positive or negative value of ΔNU represents the increase or decrease in the amount of intercropping nitrogen relative to the sole crop, respectively^20^. Phosphorus and potassium were calculated in the same way.

### Nutrient use efficiency

Nitrogen utilization efficiency is the amount of accumulated dry matter that can be produced by the unit nitrogen uptake. The intercropping nitrogen use relative to sole crop is calculated as follows:

2 $$\Delta {\rm{NUE}}=[({Y}_{ic}/N{U}_{ic})/({F}_{m}\times {Y}_{sm}/N{U}_{sm}+{F}_{s}\times {Y}_{ss}/N{U}_{ss})-1]\times 100 \% \,$$

where *Y*_*ic*_ is the yield, and ΔNUE represents the increase or decrease in nutrient use efficiency crop intercropping^20^. Phosphorus and potassium are calculated by the same method.

### Contribution of nutrient uptake and utilization efficiency to yield advantage

The LER is calculated as follows:

3 $${\rm{LER}}=\frac{{Y}_{im}}{{Y}_{m}}+\frac{{Y}_{is}}{{Y}_{s}}$$

where *Y*_*im*_ and *Y*_*is*_ are the yields of intercropped maize and soybean, respectively; *Y*_*m*_ and *Ys* are the yields of sole maize and sole soybean, respectively. For nitrogen, the uptake and utilization efficiency of maize in the intercropping and sole are *A*_*im*_, *A*_*sm*_ and *E*_*im*_, *E*_*sm*_, respectively, and that for soybeans are *A*_*is*_, *A*_*ss*_ and *E*_*is*_, *E*_*ss*_.

LER is obtained as follows:

4 $${\rm{LER}}=\frac{{A}_{im}}{{A}_{sm}}\times \frac{{E}_{im}}{{E}_{sm}}+\frac{{A}_{is}}{{A}_{ss}}\times \frac{{E}_{is}}{{E}_{ss}}$$

$${\rm{If}}:{a}_{m}=\frac{{A}_{im}}{{A}_{sm}}-1;\,{a}_{s}=\frac{{A}_{is}}{{A}_{ss}}-1;\,{e}_{m}=\frac{{E}_{im}}{{E}_{sm}}-1;\,{e}_{s}=\frac{{E}_{is}}{{E}_{ss}}-1$$

5 $${\rm{LER}}=1+(1+{{\rm{a}}}_{m}+{a}_{s})+({e}_{m}+{e}_{s})+({a}_{m}{e}_{m}+{a}_{s}{e}_{s})$$

$(1+{{\rm{a}}}_{m}+{a}_{s})$ represents the contribution of the increase or decrease of nutrient uptake caused by intercropping to intercropping yield advantage relative to single cropping; $({e}_{m}+{e}_{s})$represents the contribution of changes in nutrient use efficiency caused by intercropping to intercropping yield advantage; and $({a}_{m}{e}_{m}+{a}_{s}{e}_{s})$ represents the contribution of nutrient uptake and utilization efficiency interaction to intercropping advantage^20,40^.

### Statistical analysis

Data were analyzed using Excel software (version 2016), and statistical analysis was conducted using SPSS (version 22) and Origin software (version 2018). One-way ANOVA was employed to determine the significance of the difference among the treatments. All determinations of significance were evaluated at the probability level of 0.05.

## Supplementary information

Supplementary information.
