# Supplementary material for: Uptake and utilization of nitrogen, phosphorus and potassium as related to yield advantage in maize-soybean intercropping under different row configurations
Source: Sci Rep. 2020 Jun 11;10:9504. doi: 10.1038/s41598-020-66459-y (PMC7290029; doi:10.1038/s41598-020-66459-y)
Supplement: Supplementary file 1 — Supplementary information. [file 41598_2020_66459_MOESM1_ESM.pdf]

Uptake and utilization of nitrogen, phosphorus and potassium as related to yield advantage in maize-soybean intercropping under different row configurations

Yuanfang Fan[1, 2], Zhonglin Wang[1, 2], Dunping Liao[1, 2] Muhammad Ali Raza[1], Beibei Wang[1, 2], Jiawei Zhang[1, 2], Junxu Chen[1, 2], Lingyang Feng[1], Xiaoling Wu[1, 2], Chunyan Liu[1,2], Wenyu Yang[1, 2,3*], Feng Yang[1, 2,3*]

[1] College of Agronomy, Sichuan Agricultural University, Chengdu 611130, People's Republic of China

[2] Sichuan Engineering Research Center for Crop Strip Intercropping System, Chengdu 611130, People's Republic of China

[3] Key Laboratory of Crop Ecophysiology and Farming System in Southwest, Ministry of Agriculture, Chengdu 611130, People's Republic of China

[*]Corresponding author.

Email address: f.yang@sicau.edu.cn;

mssiyangwy@sicau.edu.cn

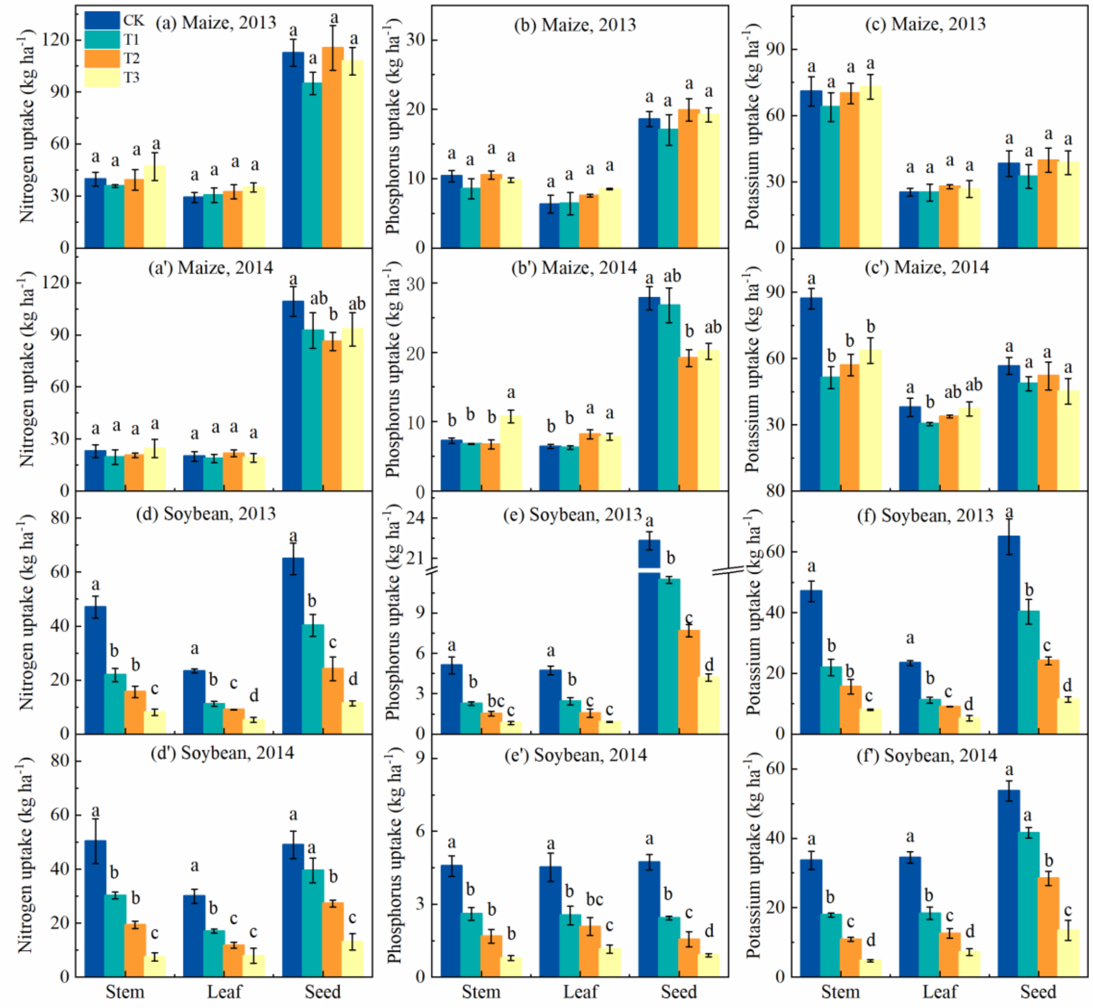

Figure S1. Nutrient uptake in stem, leaf and seed of maize and soybean under different row configurations in 2013 (a-f) and 2014 (a'-f'). The T1 (40 cm: 60 cm), T2 (80 cm: 120 cm) and T3 (100 cm: 100 cm) represent the different row configurations under the intercropping system. The CK refers to sole cropping system of maize (a-c, a'-c') and soybean (d-f, d'-f'), respectively. Means are averaged over three replicates. Bars show ± standard errors, (n = 3). Within a bar, different lowercase and same letters show a significant and non-significant difference ($p \leqslant 0.05$) between treatments.

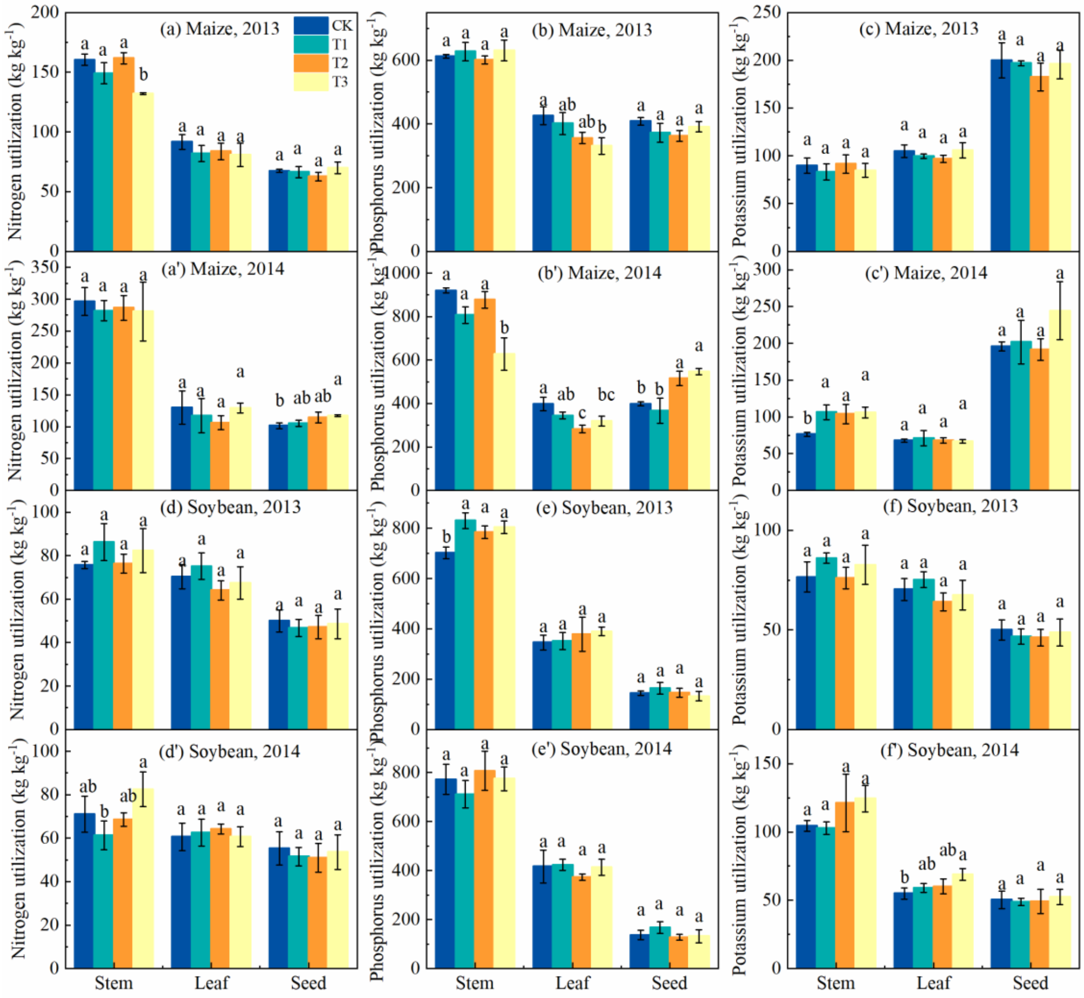

Figure S2. Nutrient utilization in stem, leaf and seed of maize and soybean under different row configurations in 2013 (a-f) and 2014 (a’-f’). The T1 (40 cm: 60 cm), T2 (80 cm: 120 cm) and T3 (100 cm: 100 cm) represent the different row configurations under the intercropping system. The CK refers to sole cropping system of maize (a-c, a’-c’) and soybean (d-f, d’-f’), respectively. Means are averaged over three replicates. Bars show ± standard errors, (n = 3). Within a bar, different lowercase and same letters show a significant and non-significant difference ($p \leqslant 0.05$) between treatments.
